# Causal Effects of Inflammatory Bowel Diseases on the Risk of Kidney Stone Disease

**DOI:** 10.7759/cureus.63230

**Published:** 2024-06-26

**Authors:** Irfan Ullah Khan, Emad Pir Rehman, Moeen Ul Haq, Dur e Nayab, Seema Shaheen, Salman Khan, Mashhood Hamid, Muhammad Salman Godil

**Affiliations:** 1 Urology, Northwest General Hospital, Peshawar, PAK; 2 General Medicine, Chinar Hospital and Dialysis Center, Abbottabad, PAK; 3 Gastroenterology, Mufti Mahmood Memorial Teaching Hospital and Gomal Medical College, Dera Ismail Khan, PAK; 4 General Medicine, Mufti Mahmood Memorial Teaching Hospital, Dera Ismail Khan, PAK; 5 Medicine, District Head Quarter Teaching Hospital/Gomal Medical College, Dera Ismail Khan, PAK; 6 Family Medicine, King Saud University Medical City, Riyadh, SAU; 7 Internal Medicine, Jinnah Postgraduate Medical Centre, Karachi, PAK

**Keywords:** ibd, risk factors, incidence rate, kidney stone disease, inflammatory bowel diseases

## Abstract

Background: Inflammatory bowel diseases (IBDs), including Crohn's disease and ulcerative colitis, have been increasingly associated with kidney stone disease, posing significant health challenges globally.

Objective: This research sought to determine the causal relationship between kidney stone disease risk and inflammatory bowel disorders.

Methodology: This retrospective cohort study included patients with IBDs, such as ulcerative colitis or Crohn's disease, who were diagnosed at least 18 years of age. Information was gathered with an emphasis on patients having comprehensive medical histories and confirmed cases of kidney stone disease from January to December 2022. Medical records were retrospectively evaluated by trained staff to extract treatment information and clinical, radiological, and demographic data. To evaluate relationships, statistical analysis was carried out in SPSS software version 23 using Chi-square tests and descriptive statistics.

Results: The study included 320 patients diagnosed with IBDs, among which 198 (61.87%) had Crohn's disease, and 122 (38.13%) were diagnosed with ulcerative colitis. The cohort consisted of 140 females (43.75%) and 180 men (56.25%), with a mean age of 45.5 years. Regarding smoking, 113 people (35.31%) reported being smokers, whereas 207 people (64.69%) did not smoke. Additionally, 18 (5.62%) of the population had an underweight BMI, 136 (42.50%) had a normal BMI, 119 (37.19%) had an overweight BMI, and 47 (14.69%) had an obese BMI. Of the patients, 86 (26.88%) had a prior history of kidney stone disease, while 194 (60.62%) did not. Aminosalicylates were the most often used therapy modality for IBD in 189 (58.97%) of cases, followed by corticosteroids in 117 (36.56%) and immunomodulators in 93 (28.94%). Radiological examinations showed that renal calculi were present in 60 (18.75%) of patients, and kidney stones occurred in 40 (12.50%) of patients throughout the research period. The smoking status (p=0.006) and prior history of kidney stones (p<0.001) were the corresponding p-values for the significant results.

Conclusion: The study highlights an increased risk of kidney stone disease in IBD patients, particularly among smokers and those with a recurrent history of kidney stones. Of the 320 patients, 198 (61.87%) had Crohn's disease and 122 (38.13%) had ulcerative colitis, with a significant relationship found between kidney stones and both smoking (113 patients, 35.31%, p=0.006) and a prior history of kidney stones (86 patients, 26.88%, p<0.001). The findings emphasize the need for targeted preventive measures and close monitoring of these high-risk groups.

## Introduction

The term "inflammatory bowel diseases" (IBDs) refers to a class of long-term inflammatory illnesses that mostly affect the gastrointestinal system, such as ulcerative colitis and Crohn's disease [1,2]. The chronic nature, difficulties, and related comorbidities of these disorders place a heavy strain on people and healthcare systems globally [3]. Among the many side effects, new research points to a possible association between IBDs and the development of kidney stone disease, a painful and recurring ailment marked by the accumulation of solid crystals in the urinary tract [4]. In order to optimize patient treatment, guide preventative initiatives, and lessen the burden associated with both disorders, it is essential to comprehend the causative impact of IBDs on the risk of kidney stone disease [5].

Recent clinical findings and epidemiological investigations have heightened interest in the connection between IBDs and kidney stone disease [6]. The exact processes causing this link are still not fully understood; however, a number of variables have been suggested as contributing to the higher risk of kidney stone disease in people with IBD [7]. Some of the hypothesized reasons that may make people with IBDs more susceptible to kidney stones include metabolic disturbances, intestinal malabsorption, chronic inflammation, and changes in the makeup of the gut microbiota [8]. Furthermore, due to their effects on calcium and electrolyte metabolism, several drugs that are often used to treat IBDs, including corticosteroids and some immunosuppressants, may further impact the development of stones [9].

There are still a number of unknowns about the relationship between kidney stone disease and IBDs. The majority of previous research has been observational, which makes it difficult to conclusively prove causation [10,11]. Furthermore, most investigations have either been underpowered to identify clinically meaningful relationships or have concentrated on certain subsets of IBD patients [12]. Therefore, to clarify the causative pathways connecting IBDs to kidney stone disease, well-designed prospective studies and mechanistic investigations are still desperately needed [13].

Research objective

The purpose of this research was to examine the relationship between kidney stone disease risk and IBD.

## Materials and methods

Study design and settings

This research employed a retrospective cohort study methodology and was conducted from January 2022 to December 2022 at Northwest General Hospital and Hayatabad Medical Complex in Peshawar, as well as Mufti Mahmood Memorial Teaching Hospital and District Head Quarter Teaching Hospital in Dera Ismail Khan, focusing on the treatment of kidney stone disease and IBDs.

Inclusion and exclusion criteria

Patients with IBDs, such as Crohn's disease or ulcerative colitis, who were 18 years of age or older were the subject of the research. For analysis, only patients with full medical records were taken into account. Throughout the designated research time, individuals had to have received therapy for their IBDs. Participants for whom demographic, clinical, laboratory, and radiological data were available were included in the research. The trial specifically focused on individuals who had kidney stone illness that had been recorded, either before or during the study period. Patients with insufficient medical records, severe renal disorders unrelated to IBDs, and kidney stone disease were not included in the research. Individuals with diseases or therapies that confounded the relationship between kidney stones and IBD were also eliminated, as was anybody who did not get treatment for IBDs throughout the research period. Furthermore, individuals who were pregnant throughout the trial period were excluded.

Sample size

In the initial phase of our research, we identified a total of 398 patients diagnosed with IBDs. However, after applying exclusion criteria, 78 patients were excluded from the final study cohort (Table 1). Several reasons, including incomplete medical records, unrelated kidney stones, severe renal conditions unrelated to IBDs, not receiving treatment for IBDs during the study period, confounding medical conditions or treatments, and pregnancy during the designated period, were the main causes of these exclusions. The "unrelated kidney stones" refer to instances where patients had kidney stones that were not attributed to or associated with their diagnosed IBD, such as Crohn's disease or ulcerative colitis. These cases include kidney stones caused by factors like idiopathic reasons, genetic predisposition, medication side effects, dietary habits, or other systemic conditions unrelated to the inflammatory processes of IBD. By excluding these cases from the sample size, the study aims to ensure that its findings accurately reflect the specific relationship between IBD and kidney stone disease, without interference from unrelated factors that could potentially confound the results.

**Table 1:** Exclusion Criteria and Number of Patients Excluded From the Final Study Cohort

Exclusion Criteria	Number of Patients Excluded
Incomplete medical records	25
Unrelated kidney stone disease	12
Severe renal conditions unrelated to IBDs	8
Lack of treatment for IBDs during study period	15
Confounding medical conditions or treatments	10
Pregnancy during the specified timeframe	8
Total	78

Data collection

Medical records of eligible patients were retrospectively reviewed to collect relevant data. Information including demographic characteristics, clinical history, laboratory investigations, radiological findings, treatment modalities for IBDs, and occurrence of kidney stone disease during the study period was extracted using a standardized data collection form. Data collection was conducted by trained research personnel to ensure accuracy and consistency.

Statistical analysis

The research population's clinical and demographic features were compiled using descriptive statistics. It was determined what proportion of individuals with IBDs had kidney stones. The study used the Chi-square test to evaluate the correlation between category variables. At p<0.05, statistical significance was established.

Ethical approval

Ethical approval (No. 267/GJMS/JC) was obtained from the Ethical Review Committee, Gomal Medical College, MTI, Dera Ismail Khan, Pakistan. Because the research was retrospective in nature and used de-identified patient data, informed permission was not required.

## Results

The research cohort included 320 IBD patients whose ages ranged across several groups: the mean age was 45.5 years (SD±13.2), with 14.38% (n=46) being between the ages of 18 and 30 years, 38.75% (n=124) being between the ages of 31 and 45, 29.06% (n=93) being between the ages of 46 and 60, and 17.81% (n=57) being beyond 60. Regarding the distribution of genders, there were 43.75% (n=140) female and 56.25% (n=180) male. Of the patients in the cohort, 61.87% (n=198) had a diagnosis of Crohn's disease, whereas 38.13% (n=122) had an ulcerative colitis diagnosis. With regard to smoking, 113 people (35.31%) reported being smokers, whereas 207 people (64.69%) did not smoke. As per Table 2, the distribution of body mass index (BMI) revealed that 18.6% of the population (5.62%) were underweight, 42.50% (n=136) had a normal BMI, 37.19% (n=119) were overweight, and 14.69% (n=47) were obese.

**Table 2:** Demographic Characteristics of Study Population *Mean±SD: 45.5±13.2 years BMI, body mass index

Characteristic		No. of Patients (n=320)	Percentage (%)
Age group*	18-30 years	46	14.38
	31-45 years	124	38.75
	46-60 years	93	29.06
	Above 60 years	57	17.81
Gender	Male	180	56.25
	Female	140	43.75
Diagnosis	Crohn's disease	198	61.87
	Ulcerative colitis	122	38.13
Smoking status	Smoker	113	35.31
	Non-smoker	207	64.69
BMI	Underweight (<18.5)	18	5.62
	Normal (18.5 to <25)	136	42.50
	Overweight (25 to <30)	119	37.19
	Obese (≥30)	47	14.69

**Figure 1:**
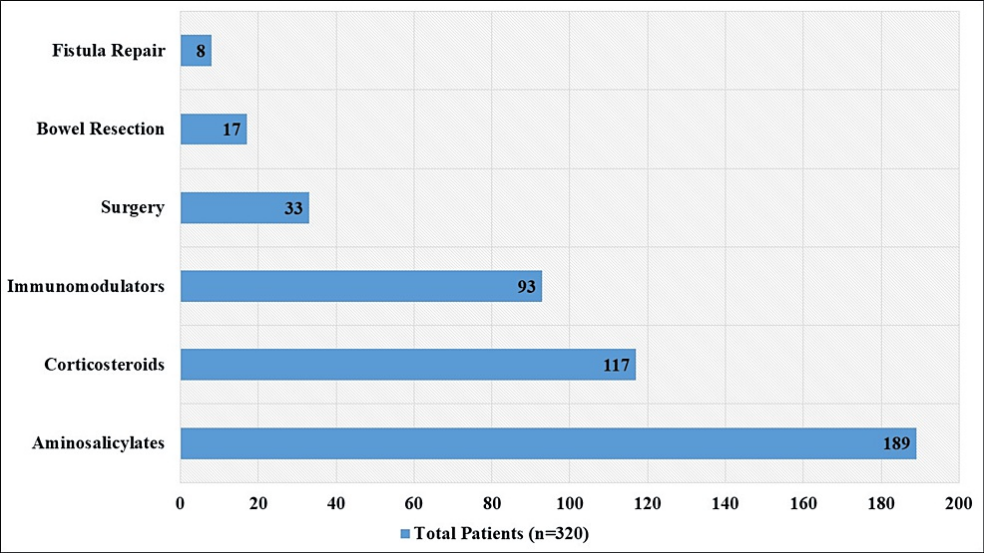
Treatment for IBDs IBDs, inflammatory bowel diseases

Different drugs were used to treat IBDs; the most often used ones were aminosalicylates (58.97%, n=189), corticosteroids (36.56%, n=117), and immunomodulators (28.94%, n=93). Surgery was less prevalent; 10.31% (n=33) of patients had surgery, with bowel resection (5.31%, n=17) and fistula repair (2.50%, n=8) among the procedures performed (Figure 1).

**Figure 2:**
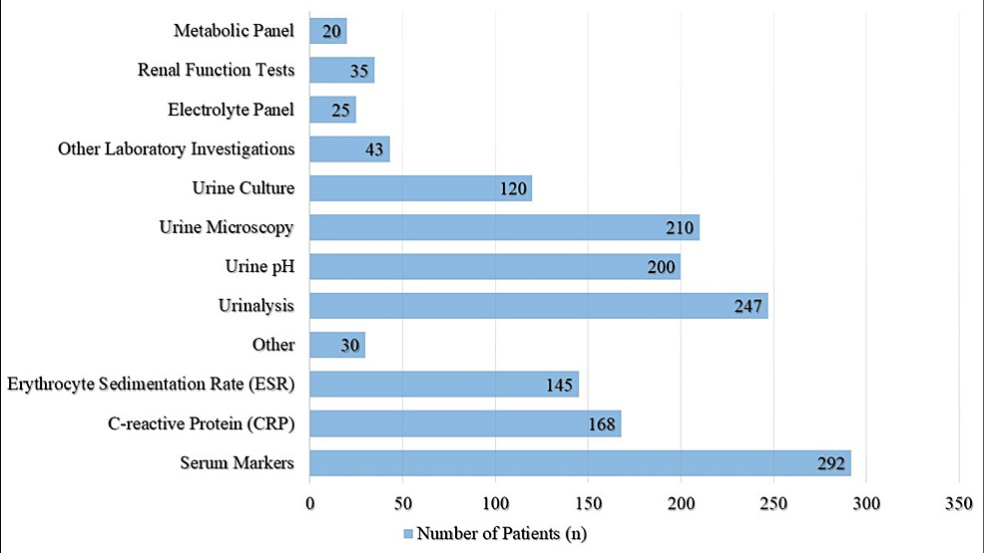
Laboratory Findings Among Patients

Extensive testing was shown by laboratory results, with 91.25% (n=292) undergoing investigation of serum markers, including erythrocyte sedimentation rate (45.31 percent, n=145) and C-reactive protein (52.50%, n=168). 77.19% (n=247) of the patients had a urinalysis, whereas 62.50% (n=200) had a urine pH test and 65.63% (n=210) had a urine microscopy. Additionally, 37.50% (n=120) of the patients had a urine culture. To provide a complete picture of the patient's health, additional laboratory tests were performed, such as metabolic panels (6.25%, n=20), renal function tests (10.94%, n=35), and electrolyte panels (7.81%, n=25) (Figure 2).

Radiological investigations were conducted in 87.50% (n=280) of cases, utilizing modalities such as X-ray, CT scan, and ultrasound for kidney stone detection (Table 3). Of these individuals, 18.75% (n=60) received results that described the kinds and locations of the stones found, which were suggestive of renal calculi. Remarkably, 12.50% (n=40) of the patients developed kidney stones throughout the research period; details on the new start or exacerbation included dates, kidney stone size, and composition. Information on the dates, frequency, and therapies for kidney stones previous to the research period was provided by 26.88% (n=86) of the participants who had a history of kidney stone illness.

**Table 3:** Radiological Findings and Kidney Stone Disease

Characteristic		No. of Patients (n=320)	Percentage (%)	Radiological Findings	Additional Information
Radiological Investigations	Imaging modalities used	280	87.50	X-ray, CT scan, ultrasound	Types of imaging modalities utilized for kidney stone detection
	Findings of kidney stones	60	18.75	Presence of renal calculi	Types and locations of kidney stones detected
Occurrence of Kidney Stone	During study period	40	12.50	New onset or exacerbation	Dates, size, and composition of kidney stones during the study period
	Prior to study period	86	26.88	History of kidney stone disease	Dates, frequency, and interventions for kidney stones before the study period

Table 4 displays the incidence rates of kidney stone disease among patients with IBDs, along with associated p-values. The incidence rate for all 320 cases was 12.50 per 100 person-years. Gender-stratified incidence rates showed that men had 15.56 cases per 100 person-years, a considerably greater rate than women (8.57 instances) (p=0.032). Nevertheless, there was no statistically significant difference in the incidence rates of Crohn's disease (10.61) and ulcerative colitis (9.83) (p=0.238). Patients between the ages of 46 and 60 had the greatest occurrence rate (19.35), followed by those between the ages of 31 and 45 (11.29), 18 and 30 (10.86), and above 60 (5.26). Even while the incidence rates differed by age group, only the 46-60-year-old group showed a statistically significant difference (p=0.128).

**Table 4:** Incidence Rate of Kidney Stone Disease Among Patients With IBDs *P-value <0.05 is significant IBDs, inflammatory bowel diseases

Group		No. of Patients (n=320)	No. of Cases (40)	Incidence Rate (per 100 Person-Years)	P-Value
By gender	Male	180	28	15.56	0.032*
	Female	140	12	8.57	
By diagnosis	Crohn's disease	198	21	10.61	0.238
	Ulcerative colitis	122	12	9.83	
By age group	18-30 years	46	5	10.86	0.128
	31-45 years	124	14	11.29	
	46-60 years	93	18	19.35	
	Above 60 years	57	3	5.26	

Table 5 outlines factors associated with kidney stone disease among patients' IBDs, along with corresponding p-values. Kidney stones were formed by 20.35% of smokers (n=23), a considerably greater rate than the 8.21% (n=17) of non-smokers (p=0.006). BMI incidence rates were as follows: 10.29% (n=14) for normal weight, 11.11% (n=2) for underweight, 13.44% (n=16) for overweight, and 17.02% (n=8) for obese people. There was no discernible difference between these incidence rates (p=0.211). Among the 320 patients studied, 40 (12.5%) had kidney stone disease. Out of those with a history of kidney stones (86 patients), 29 (33.72%) had the disease, significantly higher than the 11 (5.67%) without such history (p<0.001). Additionally, 3.2% of patients currently diagnosed had kidney stones. This suggests a strong correlation between kidney stone occurrence in the past and subsequent development in IBD patients.

**Table 5:** Factors Associated With Kidney Stone Disease Among Patients With IBDs *P-value <0.05 is significant BMI, body mass index; IBDs, inflammatory bowel diseases

Factors		No. of Patients (n=320)	Patients With Kidney Stone Disease (n=40)	Percentage (%)	P-Value
Smoking status	Smoker	113	23	20.35	0.006*
	Non-smoker	207	17	8.21	
BMI	Underweight (<18.5)	18	2	11.11	0.211
	Normal (18.5 to <25)	136	14	10.29	
	Overweight (25 to <30)	119	16	13.44	
	Obese (≥30)	47	8	17.02	
Kidney stones	History (yes)	86	29	33.72	<0.001*
	History (no)	194	11	5.67	
	Current diagnosis	40		3.2	

## Discussion

The current research set out to look at the relationship between IBDs and kidney stone disease risk. According to our research, individuals with IBDs had an incidence rate of kidney stones of 12.50 per 100 person-years overall. This incidence is in line with other studies that suggested those with IBDs had a higher chance of developing kidney stones [8]. In addition, our research revealed notable differences in incidence rates across genders, with men showing a much greater incidence of kidney stones than women. In particular, the incidence rate for men was 15.56 cases per 100 person-years compared to 8.57 instances for women. This gender disparity is consistent with previous research showing a male preponderance in kidney stone frequency in a range of populations [14-16].

Additionally, there was no statistically significant difference in the incidence rates of kidney stone disease between individuals with ulcerative colitis and those with Crohn's disease, according to our research. Particularly, kidney stone disease had an incidence rate of 10.61 cases per 100 person-years among Crohn's disease patients and 9.83 cases per 100 person-years among ulcerative colitis patients. This result is in contrast to some earlier research that suggested people with Crohn's disease had a greater incidence of kidney stones than those with ulcerative colitis [17,18]. However, variables like sample size or differences in the severity of the condition among the research group may be to blame for the study's lack of relevance.

In terms of age distribution, our findings showed that incidence rates varied throughout age groups, with those between the ages of 46 and 60 exhibiting the greatest rate. In particular, the incidence rate for individuals between the ages of 46 and 60 was 19.35 instances per 100 person-years. The overall pattern of rising kidney stone prevalence with advancing age is supported by this age-related trend [19]. It is interesting, nonetheless, that only a statistically significant difference was found between patients aged 46-60 and those over 60, despite the increased incidence rate in later age groups.

In our investigation, smoking status was shown to be a major risk factor for the formation of kidney stones in individuals with IBDs. When compared to non-smokers, smokers had a significantly higher incidence rate of kidney stones. In particular, the incidence rate for smokers was 20.35 cases per 100 person-years, compared to 8.21 instances for non-smokers. In our investigation, smoking status was shown to be a major risk factor for the formation of kidney stones in individuals with IBDs. This result is in line with other studies that found a favorable correlation between smoking and the development of kidney stones, attributing it to changes in renal blood flow, decreased urinary citrate, increased urinary calcium, and a higher excretion of oxalate, all of which contribute to stone formation [20].

Furthermore, among patients with IBDs, our research found a significant correlation between the incidence of recurring kidney stones and a prior history of the condition. Individuals who previously had kidney stones had a much greater incidence rate than those who had not. In particular, the incidence rate for individuals with a history of kidney stones was 33.72%, while the rate for those without a history was 5.67%. This result emphasizes how crucial it is to keep an eye on and treat kidney stone recurrence in this particular cohort. It highlights the clinical importance of a thorough evaluation of the patient's medical history in anticipating and averting recurrent kidney stone events in people with IBDs [21].

The current study on the relationship between IBDs and kidney stone disease risk aligns with previous research indicating higher incidence rates of kidney stones in individuals with IBDs, and it highlights gender differences consistent with established patterns of higher male prevalence. Contrary to some studies suggesting a higher incidence of Crohn's disease than ulcerative colitis, our findings show no significant difference, possibly due to sample size or disease severity variations. Age-wise the incidence peaks in the 46-60 age group, supporting age-related trends in kidney stone prevalence. Our study corroborates previous findings on smoking as a significant risk factor and underscores the importance of monitoring and managing recurrent kidney stones in IBD patients, given the notably higher recurrence rates in those with a prior history of stones.

Limitations

The sample size and focus on specific healthcare facilities may limit the generalizability of the findings to broader populations. The observational nature of the study precludes causal inference, and reliance on retrospective medical records may introduce variability and bias. Despite these constraints, our study offers valuable insights into this understudied association, highlighting the need for further research to elucidate the underlying mechanisms and implications for clinical practice.

## Conclusions

This study underscores the heightened risk of kidney stone disease among individuals with inflammatory bowel disorders, emphasizing the imperative for tailored preventative measures and vigilant monitoring to mitigate this burden. The findings reveal significant associations between smoking status, prior history of kidney stones, and the occurrence of renal calculi. As the prevalence of both IBDs and kidney stone disease continues to rise globally, proactive management and targeted interventions are essential to improve patient outcomes and alleviate the impact on healthcare systems.
